# Ethnomedicinal plant use by Lepcha tribe of Dzongu valley, bordering Khangchendzonga Biosphere Reserve, in North Sikkim, India

**DOI:** 10.1186/1746-4269-4-22

**Published:** 2008-10-01

**Authors:** Bharat K Pradhan, Hemant K Badola

**Affiliations:** 1 Conservation of Biodiversity Core Group, G.B. Pant Institute of Himalayan Environment & Development, Sikkim Unit, P.O. Box 24, Gangtok 737 101 (Campus at Pangthang), Sikkim, India

## Abstract

Lepcha is the oldest and the first tribe reported from Sikkim, India; majority of its population inhabiting in Dzongu valley, an officially demarcated reserve for Lepcha community, bordering Khangchendzonga Biosphere Reserve, in north district. Lepchas of Dzongu are known for their retention of rich cultural heritage. In view of the on-going cultural and economic changes brought in by the process of globalization, the immediate need was felt to document in details the under-explored ethnomedicinal practices of Lepchas of Dzongu valley. This paper reports 118 species, belonging to 71 families and 108 genera, under ethnomedicinal utility by the Lepchas for curing approximately 66 ailments, which could be grouped under 14 broad categories. Zingiberaceae appeared as the most used family (8 species and 5 genera). As per use pattern, maximum of 30.50% species are to cure stomach related disorders/ailments, followed by 19.49% for curing cut, wounds, inflammation, sprains and joint pains. Administration of medicine orally is recorded in 75% cases. Root and rhizome harvesting targeted 30 species. The changing scenario over time both at socio-cultural front and passing traditional knowledge interests from older to younger generation and rich ethnomicinal wealth of the oldest tribe of Sikkim are discussed in the light of conservation strategies and techniques to adopt.

## Introduction

Documentation of traditional knowledge on ethno medicinal use of plants has been considered as a high priority [1-5] to support the discoveries of drugs benefiting mankind. In India, various communities use over 50% of the plant species of any ecosystem in ethnomedicine and in general over 7500 species are utilized in primary health care by various tribes [6]. The tribal populations, who have been the primary inhabitants of natural habitats, hold tremendous amount of traditional knowledge on the use of various biotic resources [4,7], which may have greater importance to the on-going research and discoveries in the field. It is well acknowledged in literature [2,8,9] that their age old practices of using plants to cure numerous ailments have paved the way to further discovery of many life saving drugs. In India, out of over 427 tribal communities in total, the north-east states, including Sikkim, boost to have over 130 major tribes, reaching in to a total sub-tribes or groups of about 300 [10]. The state of Sikkim, though only 7096 Km^2 ^in area is one of the rich depositories of biota [11]. This represents over 550 medicinal plants, which may offer incredible scope for the development of pharmaceutical sector as potential commercial hub, boosting economy of the state. Ethno-medicinal explorations and simultaneous prioritization of pharmaceutically important plant species for conservation through ex-situ cultivation have been identified as vital aspects for the drug industrial development [6,12,13]. In Sikkim, such exploratory researches on ethnomedicinal use of plants are not sufficiently taken up, especially targeting the remotely located tribal areas in the state; whichever is available mostly confined to simple preliminary listings (mentioned later in this section). The Dzongu valley in north Sikkim, India inhabits the largest population of  the Lepcha tribe. The Dzongu valley, an officially demarcated reserve for Lepcha community bordering Khangchendzonga Biosphere Reserve, known for its vast plant wealth is one of the least attended areas on ethnomedicinal aspects, for being sacred and restricted, especially to outsiders.

The Lepchas of Dzongu valley, an isolated forest dweller, living harmoniously with nature over centuries, have accumulated a vast understanding on the use pattern of various wild products of the area. This suggests them as great traditional ethno-botanical practitioners. Sir J.D Hooker, during his botanical explorations to Sikkim Himalaya (1847–1851), mentioned Lepchas of Sikkim for their knowledge on the plants in splendid terms in his monumental work,"Himalayan Journal (1855)" [14]. The bamboo plant has been used quite commonly and exquisitely by the Lepcha community since their existence. Bamboo supplies a frame in the majority of constructions, such as houses and bridges. Lepchas seem to have gained marvel over the technical use of Bamboo, ranging from articles of routine requirement to artifacts, water distribution network, musical instruments, etc [15]. Bamboo is a summom bonum of their spirit. The Bamboo technology can be imported from this community. Lepchas in remoteness from modern facilities of the world got adapted to develop skills required to withstand difficult conditions of nature. Lepchas have become carrier of enormous understanding on the use of plants descended upon them through use of traditional medicinal cure to various ailments [16]. However, a general observation highlights that the Lepcha medicine man or the Lepcha healer, locally called 'Maon-doak', is known to restrict his medicinal practices and prescriptions only to Lepcha community, and he does not share or offer the same to the outsiders. The 'Maon-doak' believes that if his secret traditional knowledge of using plants is disclosed to any unauthorized person, the plants under use would produce adverse effects, and he may encounter ill-fate generated from the rage of the supreme deity of medicinal plants in the forest. This non-sharing attitude must have been one of the strongest reasons for the decline of this archaic system of medicine [14].

The cultural heritage of Lepcha tribe of Sikkim has been in the past and now a centre of attraction for several anthropological studies [15-25], as well as on Lepcha language, heritage and culture in general (; accessed on 17.4.2008, for a detailed compiled list of references) but on ethnomedicinal knowledge of Lepchas only a few sporadic publications are available documenting fragmentarily. Amongst them, as a part of ethnobiological study, Jana & Chauhan [26] have tabulated the use of 38 plant species curing various ailments by Lepchas in Dzongu, giving the plant name, part used, application, etc. Similarly, 21 species of medicinal plants used (part, specific use, and doses) by both Lepchas and Nepalese in north Sikkim, in general, were reported by Maiti et al [27], who further showed concern on the regular collection of plants by the Nepalese collectors. Jha et al [28] have provided names of 35 drug plants (no individual use of plant given), and 15 local name of drug plants are mentioned, without providing botanical name, used in Dzongu. Misra & Dutta [29], in a report on Sikkim, tabulated thirty eight plants for Lepcha's folk medicine, using secondary source [26]. In a conference Abstract, Jha et al [30] have figured out 56 medicinal plants, without mentioning them, for north Sikkim. Out of above few fragmentary reports, merely 30–50 species having medicinal importance to Lepchas of Dzongu could be drawn. There are, however, numerous plant species said to be used by Lepchas in their traditional medicine which need systematic investigations and exploration. The literature lacks written records on Lepcha medicine which could have otherwise been served as the guide to the people interested in indigenous medicine [31]. Since, the Lepchas of Dzongu are known for their retention of rich cultural heritage, and especially in view of the on-going cultural and economic changes brought in by the process of globalization, the immediate need was felt to document in details the under-explored ethnomedicinal practices of Lepchas of Dzongu valley. The present study makes an exhaustive effort in investigating and documenting ethnomedicinal plants of Dzongu. The paper extended the list of such species describing their detailed practices along with quantitative analysis of the data. This study will present an updated and much improved document of the traditional pharmaceutical knowledge of a tribe of Dzongu valley. This effort should be seen serving not only as a sound base for resource assessment but an opportunity for developing scientific guidelines on access and benefit sharing regime on ethnomedicinal plants by the community people. The objectives of the present study is to provide field based assessment and documentation on, (i) authentic listing of plants used in traditional medicinal practices; (ii) the use part and the use pattern of the plants, preparation, ailments cured, etc., and (iii) describing conservation aspects of those plants for the drug.

### The Lepcha tribe- a brief history

The Lepcha tribe is believed to be the indigenous to Sikkim Himalaya [18,25]. This tribe claims to have its origin in the "Ne Meyel Lyang" (the land of hidden paradise), or "Ne Male Lyang" (land of internal purity), a legendry kingdom on the slopes of Khangchendzonga mountain comprising Sikkim, and Ilam hills, now in Nepal [32]. The Lepchas are characterised by Mongoloid morphological features [18]. However, according to White [33], Lepchas came from the eastern direction of Assam and Burma and settled in Sikkim. He further menioned that, the Lepchas believed to have similarity with the Tibetans, but Tibetans are smaller and slighter in built with finer cut features, and in many cases the Lepchas are almost like Jewish. The Lepchas have resemblance with the tribes of Hanga-rang in the North West Frontier Province and also with the mountain tribes of the Laree area in Ladakh. Some also believed that the Lepchas were originated in China and belong to Ta-Tai group of Chinese [23]. The union of two words *lep *and *tsa *means 'to belong to a place' coins the word Lepcha as originated [34]. In connection with origin of the word "Lepcha", Risley [35] writes ".........*what the derivation of Lepcha is cannot be ascertained. It must, however, be remembered – that the English form of spelling the word is incorrect and out of keeping with the local pronunciation, which is "Lap-cha" or "Lap-che," the former being the more common and probably the correct one. Dr. Waddell writes: "As the term' Lapcha' is of Nepalese origin, and the Parbatiya dialect of the Nepalese consists mainly of pure Sanskrit roots, the word 'Lapcha' may perhaps be derived from' lap,' speech, and' cha,' vile = the vile speakers-a contemptuous term with reference to their" non-adoption of the Parbatiya language like the rest of the' Nepalese' tribes." Another authority enquires whether it may refer to the Hindi, Lap-thi,' the name of a kind of skate fish, i.e., of a flat fish, a term which may have been applied by the Goorkhas to the Lepchas on account of the flatness of their faces. None of these derivations are convincing, but none are offered by the people themselves*............". The distinct Lepcha language known as "Rong" [36], belonging to Tibeto-Kanauri group, included in Tibeto-Burman family of languages, is distinguished by having its own script (supposed to be invented by the Lepcha scholar Thikúng Men Salóng sometimes during the 17^th ^century) and literature [33]. Lepchas indentify themselves as "Rong-kup" meaning the 'son of snowy peak' [24], "Rong-Pa" meaning 'Ravine folk or the dwellers of the valley' [17,34], and "Mutanchi" meaning 'beloved people of mother earth'.

The Lepchas were hunters and gatherers [21,34] and used to live complete nomadic lives. Since mid-nineteenth century, they began practicing settled agriculture [37] particularly because of increased production of large cardamom, as a cash crop. In addition, Lepchas also grow rice, maize, millet, wheat, buckwheat, pulses, and vegetables, and in some parts sugarcane and fruits, with animal husbandry as another important economic activity. The diet of Lepchas is supplemented with plants and mushrooms, tubers and rhizomes gathered from wild and produce grown in small kitchen garden such as ginger, chilies, beans, cucumber, garlic, sweet potatoes, yams and sugarcane. Originally, the Lepchas were the followers of the Shamanism; they converted to Bhuddism in eighteenth century, and since the middle of the nineteenth century, a significant number of Lepchas has converted into Chiristianity [38]; although, indigenous Lepcha Shamanism has managed to exist till today.

### Study area and methodology

#### Study area

An officially demarcated reserve for Lepcha community, the Dzongu, a Bhutia derived name meaning "a place with nine districts" [19], is located about 70 km north to the State Capital, Gangtok – in the north district of Sikkim, India. The Dzongu is bounded to the south-east by Teesta river and north-east by Tholung chu (river) and to the west by rising mountain leading to Khangchendzonga, the house of five treasures ['Kingtsoom Zaongboo Choo", Lepcha name for Mt. Khangchendzonga (3^rd ^highest mountain in the world) meaning 'bright auspicious forehead peak' that borders the Khangchendzonga Biosphere Reserve (KBR) at north. The KBR was officially notified in the year 2000, covering 2, 61,992 hectares area; the Dzongu valley people have traditional association with the reserve for their resources and religious affinity, and currently promoted eco-tourism by the state government. A fairly triangular shaped Dzongu landscape covers approximately 78 km^2 ^geographical areas extending between 27°28' – 27°38' N lat. and 88°23' – 88°38' E long. (as judged from Google Earth) along the 700 m to 6000 m amsl altitude. Dzongu further extends from Sheep-Gyer in the east to Sakyong-Pentong village in the west and Kishong Cho Lake in north to Lum village in the south. The area is characterized by diverse snowy mountainous landscape with steep and narrow valleys and gorges with well drained flanking slopes, receiving high rainfall between June and September. Owing to dense forest cover, the area experiences showers almost throughout the year. The area represents three climatic zones viz. sub-tropical, temperate and alpine. Further, the area may be divided into two parts, viz. Upper Dzongu; the western side of which can be entered through a bridge at Sankalang over river Teesta and the eastern side is connected by road at Theng via Toong prior to reaching Chungthang; and the Lower Dzongu, which can also be entered through a bridge at Sankalang in the eastern side and a bridge at Phedang near Dikchu bazaar (market) in the western side over the same river. Dzongu is the abode of majority of Lepchas [21]; however, as per 2004 official list of voters, it has a total population of approximately 4513 persons (*ca*. 10% of total Lepcha population of Sikkim), spreads over 38 villages.

The importance of Dzongu valley is further enhanced by the famous Tholung Gumpa, one of the oldest monasteries in Sikkim built in early 18^th ^century during the reign of Chogyal Chagdor Namgyal, the king. The Gumpa is situated at an altitude of 2600 m amidst sacred groove "a treasure house of nature", demarcated under buffer zone III of the Khangchendzonga Biosphere Reserve. Since the invasion of Sikkim by Gorkhas during late 17^th ^and early 19^th ^century, Tholung Gumpa (monastery) harbours sacred Buddhists and Sikkim relics that were brought here for safety by Lama Lhatsun Chempo, founder of the Tholung monastery. The Ecclesiastical Department, Government of Sikkim keeps these relics in sealed thirteen boxes under custody. In the presence of lamas of the Gumpa and the representatives from concerned department, these relics are taken out once in every three years in the month of April for public display. Tholung Gumpa has very sparse human habitation with merely 15–20 settlements of which 10–12 remain almost vacant throughout the year. The Kishong Cho or Kishong Lake, situated at an altitude of approximately 4200 m having religious significance for Buddhists, also forms a part of Dzongu valley. In addition, there are many sacred caves in Dzongu valley which are said to be used by the lamas for meditation in the past. Large Cardamom cultivation is practiced in the entire Dzongu valley, reaching up to Tholung Gumpa. Both in composition and value, the floristic wealth of Dzongu and its surrounding area is rich and diverse, representing a wide variety of tree species, shrubs, lichens, epiphytes, mosses and bamboos, which provides refuge to several endangered species of birds and animals. Without prior permission from the higher authorities, outside people are not allowed to visit Dzongu valley, being a restricted area [26].

#### Medicinal plant survey

At first, extensive literature and internet search was carried out to review and assess the existing information on the medicinal plants used by Lepcha tribe, as baseline for extensive research. To get first hand data and further for confirming authenticity of the existing information, extensive field surveys were undertaken between 2006 (groundwork) and 2007 (comprehensive) in Dzongu area, North Sikkim, India. Information was gathered, using semi-structured formats, interviews, and group discussions, on the indigenous uses of plant species as medicine by the Lepcha tribe. During the survey period, conversations with informants were held with the assistance of local resource persons. In view of their belief not to share their knowledge to the outsiders the collection of information was not easy. The objectives of the study were elaborated and efforts were made to take them into confidence that purpose of this study is just to document and preserve the traditional knowledge of Lepchas on medicinal plants. In total 125 informants (95 males and 30 females) were interviewed, which included 4 Moandoaks (Lepcha medicine man or Lepcha healer), 27 Thyongs (elderly person in village), 7 Bongthings or Padem (priest following Shamanism), 3 Monks, 2 Muns (a healer who exorcises demons, helps to heal illness and guides souls to the afterlife), 17 graziers and remaining 65 included people belonging to different categories like villagers, farmers, housewives, teachers, shopkeepers, forest managers, contractors, etc., of which 37 were males and 28 were females in different study villages (Passingdang, Lingdem, Fourth-mile hamlet, Ruk Lu, Kayeem, Tingvong, Tholung Gumpa, Sakyong-Pentong). These informants were approached and requested to share their knowledge about the plants they use against different diseases, plant parts harvested, method of preparation, etc. All the informants were above 27 years in age. Friendly chats made with teenagers and youngsters and school children, of both genders, helped a lot in confidence building with tribal people. In some villages, the informants were not much cooperative to reveal the secret of their ethnomedicinal knowledge to the strangers unless they were taken in to confidence, which experienced rather as a difficult task, besides language problem. Adopting participatory and group interaction approach, data were further cross-checked. Surveys were also made in the wilderness along altitudinal transects reaching timber line zones (upto Temreng), surrounding natural habitats and the agricultural areas of villages. The help of local representatives was taken to approach the plants growing in areas and or specimens available in the villages with elderly people in some cases. Species were identified using standard Floras and books [9,39,40]. The restriction on the collection of any specimens, especially by the outsiders, for being the landscape as protected/sacred/restricted, suggested adopting the above strategy of field identification. The gathered field information was systematized and analyzed to draw a clear and updated picture of the ethnomedicinal use pattern of plants of Dzongu area in Sikkim. At the same time, efforts were made to compare and discuss the use of some of the medicinal plant species recorded in Dzongu valley with those reported for other tribal groups/traditional healers in India (Table 1).

**Table 1:** Comparison on the use of some of the medicinal plant species by the different tribal groups/traditional healers in India

**Species**	**Lepcha Tribe** **[Present study]**	**Apatani Tribe **[10]	**Jaintia Tribe **[47]	**Tolcha Bhotiya**[48]	**Paliyar Tribe **[49]	**Traditional**[50]
*Acorus calamus *Linn.	**Part use: **Root/Rhizome **Disease: **Skin diseases, fever, cough	**Part use: **Root/Rhizome **Disease: **Cuts, wounds. skin diseases, bone fracture	**-----**	**-----**	**Part use: **Root/Rhizome **Disease: **Throat infection	**Part use: **Root/Rhizome **Disease: **Throat infection
*Ageratum conyzoides *Linn.	**Part use: **Leaf **Disease: **Cut, wounds, diarrhoea, dysentery, intestinal colic with flatulence	**Part use: **Leaf **Disease: **Cuts, wounds	**Part use: **Leaf **Disease: **Cuts, wounds	-----	-----	-----
*Allium cepa *Linn.	**Part use: **Bulb **Disease: **Fever, act as cooling agent	**Part use: **Bulb **Disease: **Eye pain	**-----**	**-----**	**-----**	**-----**
*Bischofia javanica *Blume	**Part use: **Leaves, bark **Disease: **Sore throat, diarrhoea	**-----**	**-----**	**-----**	**Part use: **Bark **Disease: **Nervous disorder, to stimulate hair growth	**-----**
*Cannabis sativa *Linn.	**Part use: **Seed **Disease: **Body ache	**-----**	**-----**	**Part use: **Seed, leaf **Disease: **Burn and muscular pain, stomach pain, worms	**-----**	**-----**
*Citrus aurantifolia *Christum.	**Part use: **Root, fruit, seeds, leaf **Disease: **Worms, vomiting sensation	**-----**	**-----**	**-----**	**-----**	**Part use: **Leaf **Disease: **Fever, headache, cold
*Coriandum sativum *Linn.	**Part use: **Shoot **Disease: **Expelling gas, indigestion, stomach pain	**-----**	**Part use: **Fruit **Disease: **Stomach pain	**-----**	**-----**	**-----**
*Costus speciosus *Smith	**Part use: **Root/Rhizome **Disease: **Veneral disease, urinary tract infection	**-----**	**-----**	**-----**	**Part use: **Leaf **Disease: **Diabetes	**-----**
*Curcuma aromatica *Salisb.	**Part use: **Root/Rhizome **Disease: **Nausea, stomach ache, expelling gas	**Part use: **Whole plant **Disease: **Blood purification	**-----**	**-----**	**-----**	**-----**
*Curcuma caesia *Roxb.	**Part use: **Root/Rhizome **Disease: **Expelling gas	**Part use: **Root/Rhizome **Disease: **Cough, asthma	**-----**	**-----**	**-----**	**-----**
*Curcuma longa *Linn.	**Part use: **Root/Rhizome **Disease: **Throat pain, cold, cough, fever	**-----**	**Part use: **Root/Rhizome **Disease: **Dyspepsia	**-----**	**-----**	**-----**
*Curcuma zedoaria *Roxb.	**Part use: **Root/Rhizome **Disease: **Skin disease, diarrhoea and colic, indigestion	**Part use: **Root/Rhizome **Disease: **Cold, cough	**-----**	**-----**	**-----**	**-----**
*Cynodon dactylon *(Linn.) Pers.	**Part use: **Leaves, root **Disease: **Piles, cuts, wounds, diarrhoea, dysentery	**-----**	**-----**	**-----**	**-----**	**Part use: **Whole plant **Disease: **Cooling agent for body
*Dillenia indica *Linn.	**Part use: **Leaves, fruit **Disease: **Fever, constipation, dysentery	**Part use: **Fruit **Disease: **Stomachache	**-----**	**-----**	**-----**	**-----**
*Dioscorea alata *Linn.	**Part use: **Tuber/Rhizome **Disease: **Throat pain	**Part use: **Tuber/Rhizome **Disease: **Indigestion	**-----**	**-----**	**-----**	**-----**
*Drymaria cordata *Willd. Ex Roem & Schult	**Part use: **Whole aerial part **Disease: **Sinusitis and nose blockade, headache, sore throat pain, fever, headache	**-----**	**-----**	**-----**	**Part use: **Leaf **Disease: **Cracked heel	**-----**
*Fagopyrum esculentum *Moench.	**Part use: **Grains **Disease: **Diarrhoea	**-----**	**-----**	**Part use: **Leaf **Disease: **Headache, fever	**-----**	**-----**
*Ficus hirta *Vahl.	**Part use: **Root **Disease: **Food poison	**Part use: **Fruit **Disease: **Cuts, wounds	**-----**	**-----**	**-----**	**-----**
*Ficus religiosa *Linn.	**Part use: **Whole plant/bark/fruit **Disease: **Burning sensation of genitals, vomiting, cracked heel	**-----**	**-----**	**-----**	**-----**	**Part use: **Leaf **Disease: **Body pain
*Juglan regia *Linn.	**Part use: **Bark **Disease: **Worms	**-----**	**-----**	**Part use: **Seed, bark **Disease: **Body itching, stomachache	**-----**	**-----**
*Lantana camara *Linn.	**Part use: **Leaves **Disease: **Cuts, wounds, pain reliever	**-----**	**-----**	**-----**	**-----**	**Part use: **Flower **Disease: **Headache
*Mimosa pudica *Linn.	**Part use: **Root **Disease: **Piles, boils	**-----**	**Part use: **Root **Disease: **Piles	**-----**	**-----**	**Part use: **Leaf **Disease: **Cuts, wounds
*Momordica charantia *Linn.	**Part use: **Fruit, tender shoot/root **Disease: **Diabetes, blood purification, snake bite	**-----**	**Part use: Leaf** **Disease: **Rabies, chest/rheumatic pain	**-----**	**-----**	**-----**
*Musa paradissica *Linn	**Part use: **Sap **Disease: **Fever	**Part use: **Fruit **Disease: **Indigestion	**-----**	**-----**	**-----**	**-----**
*Oroxylum indicum *(L.) Kurz	**Part use: **Bark, seed **Disease: **Fever, pneumonia	**Part use: **Seed **Disease: **Headache	**-----**	**-----**	**-----**	**-----**
*Oxalis corniculata *Linn.	**Part use: **Whole plant **Disease: **Appetizer, boils, dysentery, throat pain	**Part use: **Shoot **Disease: **Appetizer, headache	**-----**	**Part use: **Leaf, root **Disease: **Cuts, wounds	**-----**	**-----**
*Picrorhiza kurrooa *Benth.	**Part use: **Root/Rhizome **Disease: **Fever, cough	**Part use: **Root/Rhizome **Disease: **Fever, cold	**----**	**Part use: **Root/Rhizome **Disease: **Jaundice, stomachache, dyspepsia, dysentery	**-----**	**-----**
*Rhododendron campanulatum *D. Don	**Part use: **Leaves **Disease: **Cough	**-----**	**-----**	**Part use: **Leaf **Disease: **Cuts, wounds, cold, cough	**-----**	**-----**
*Rubia cordifolia *Roxb. Ex Fleming	**Part use: **Root **Disease: **Urinary infection, skin diseases	**Part use: **Root **Disease: **Cracked heel	**-----**	**-----**	**Part use: **Shoot **Disease: **Stomachache	**-----**
*Rubus ellipticus *Smith.	**Part use: **Tender shoot, root **Disease: **Stomach pain, worms, headache	**Part use: **Fruit **Disease: **Indigestion	**-----**	**Part use: **Root **Disease: **Stomachache	**-----**	**-----**
*Rumax nepalensis *Sreng.	**Part use: **Whole plant **Disease: **Wounds, hair loss	**Part use: **Leaf **Disease: **Indigestion	**-----**	**Part use: **Leaf **Disease: **Indigestion	**-----**	**-----**
*Urtica dioica *Linn.	**Part use: **Whole plant **Disease: **Bone fracture and dislocation, diarrhoea, cough, child delivery	**Part use: **Leaf **Disease: **Bone fracture	**-----**	**-----**	**-----**	**-----**
*Zingiber officinale *Rose.	**Part use: **Rhizome **Disease: **Cough, fever, throat pain	**Part use: **Rhizome **Disease: **Cough	**-----**	**-----**	**-----**	**-----**

## Results

**Figure 1:**
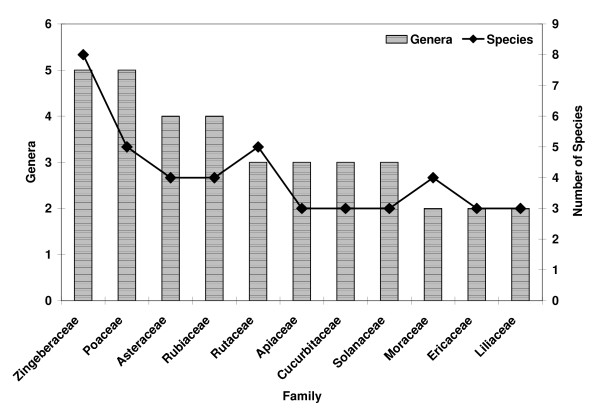
Dominant families of medicinal plant species used in Dzongu valley, North Sikkim, India.

**Figure 2:**
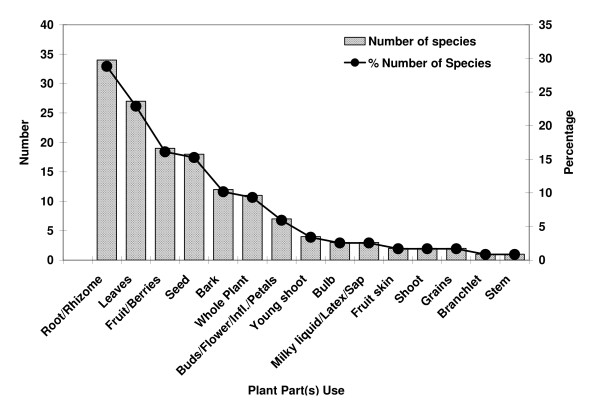
Frequency of plant parts used of medicinal plant species in Dzongu valley, North Sikkim, India.

The study documented 118 medicinal plant species, distributed across 71 families and 108 genera, used by the Lepcha tribe of Dzongu area (Table 2). In terms of number of species used, Zingiberaceae appeared as the most prominent family (8 species, 5 genera), followed by Rutaceae and Poaceae (5 species each), Asteraceae, Rubiaceae, Moraceae (4 species each), Apiaceae, Cucurbitaceae, Solanaceae, Liliaceae, Ericaceae (3 species each) (Figure 1). As per plant part used by Lepcha tribes for ethnomedicine, the maximum number of species are harvested for root and rhizome (34 species combined) and leaves (27 species), followed by fruit, seed, bark and whole plant (Figure 2). Further, destructive harvesting for the whole plant as medicine indicates the use of 9.32% species in the area. In the present study, a maximum of about 29% species are subjected to destructive harvesting using root/rhizome, which may be related to their possible vulnerability towards endangerment [41]. The cases of *Aconitum ferox, A. heterophyllum, Picrorhiza kurrooa, Swertia chirayita, Valeriana hardwickii, etc*. appeared in the same category.

**Table 2:** Plant species used for curing different ailments by the Lepcha tribe of Dzongu valley in North Sikkim, India

**S. No.**	**Botanical Name**	**Family**	**Parts used and the methods**
1	*Abies densa *Griff.	Abietaceae	Fresh leaves Juice is taken orally to relieve stomach pain and fever.
2	*Aconitum ferox *Wall ex Ser.	Ra nunculaceae	Rhizome, extremely poisonous, is detoxified by continuous boiling with water for 24 hours or more and then cut into small pieces and dried. Dried pieces are chewed to cure cough, fever, skin diseases and to relieve gout pain.
3	*Aconitum heterophyllum*	Ranunculaceae	Rhizome is dried up and taken to relieve body-ache, fever, cold, cough, nose discharge etc.
4	*Aconitum spicatum *Stapf.	Ranunculaceae	Rhizome is detoxified by non-stop boiling with water at least for 24 hours, and cut into small pieces and dried, and chewed in case of food poisoning, diarrhoea, cough, inflammation of intestine. Dried rhizome is powdered and consumed to relieve body pain, ear and nose discharge.
5	*Acorus calamus *Linn.	Araceae	External application of rhizome paste cures skin diseases and on the forehead in case of fever. Small piece of dried rhizome is taken curing distressing cough. Dried cut piece is given to child for speech clarity or to stammering child.
6	*Aesandra butyracea *(Roxb.) Baehni	Sapotaceae	Fruit juice applied on the body before sleeping to soften skin; fruit edible.
7	*Ageratum conyzoides *Linn.	Asteraceae	Leaf juice is applied externally to heal surface wounds. Decoction of herb is also given to cure stomach ailments such as diarrhoea, dysentery and intestinal colic with flatulence
8	*Allium cepa *Linn.	Liliaceae	Eating raw bulbs eaten raw reduces fever acting as cooling agent.
9	*Allium sativum *Linn.	Liliaceae	Raw bulbs are taken in case of indigestion and altitude sickness. Bulb paste cures skin diseases, and the bulb juice is poured in the ear to treat earache. Bulb fried with mushroom act as antidote on snake bite. To drive the snake away from the vicinity of the house during summer months, the rhizome is crushed to mix into with water to sprinkle around the house.
10	*Amaranthus tricolour *Linn.	Amaranthaceae	Curry prepared from green leaves stops diarrhoea. Seeds grounded into powder, mixed with water and taken as an infusion to cure general gastric problems. Beaten seeds are fried with butter and fed to pregnant women to lessen pregnancy pains.
11	*Amomum subulatum *Roxb.	Zingeberaceae	Gargle with seed decoction with water, is used to treat teeth and gum infection. Pounded root mixed with water treats urinary infection in cattle.
12	*Ampelocissus sikkimensis *(Laws) Planch.	Vitaceae	Plant juice cures sores in mouth and tongue of an infant, and treats foot and mouth disease in cattle.
13	*Artemesia vulgaris *Linn.	Asteraceae	Crushed leaves inserted in the nose stop bleeding. Water, mixed with crushed leaves, in taking bath prevents and cures allergy. Raw leaves chewed are good for mouth ulcer; also find uses in rituals.
14	*Bauhinia variegata *Linn.	Caesalpiniaceae	Dried buds are chewed to cure ulcers and bleeding piles. During toothache bark juice is taken in the form of tonic.
15	*Bergenia ciliata *(Haw.) Sternb.	Saxifragaceae	Crushed rhizome is tied around the fractured bone to heal; the paste is applied on the cuts and wounds.
16	*Bischofia javanica *Blume	Bischofiaceae	Chewing raw leaves treat sore throat. Drinking bark cure diarrhoea.
17	*Brassica campestris *Linn.	Brassicaceae	Seed oil is applied to wounds to speed up healing and prevent infection. Oil applied on forehead relieves headache. To keep hair black and healthy, the oil is applied with massage.
18	*Calamus macracanthus *T. Anders.	Arecaceae	Juice of crushed leaves used as eye drop cures eye infection and other eye diseases.
19	*Canna indica *Linn.	Cannaceae	Edible rhizome is boiled and taken during fever.
20	*Cannabis sativa *Linn.	Urticaceae	Pounded seeds mixed with water taken in very minute quantity during severe body pain; the leaves given to cattle in flatulence.
21	*Carica papaya *Linn.	Caricaceae	Raw fruit is crushed, squeezed and the milky extract given to females for aborting unwanted pregnancy.
22	*Cedrela toona *Roxb.	Meliaceae	Bark is crushed and the paste is applied to cure ulcers. Flower is chewed to promote menstrual discharge in females.
23	*Celastrus paniculatus *Willd.	Celastraceae	Seed paste is applied in case of skin irritation/allergy; good for gout.
24	*Cinnamomum tamala *(Buch.-Ham.) Nees. & Eberm.	Lauraceae	Leaves are rubbed on the body surface of the scabies affected person.
25	*Cissampelos pareira *L.	Menispermaceae	Plant extract is given to treat diarrhoea, dysentery, indigestion and urinary disorders. Root is used as antidote. Leaves applied on wounds heal and cure stomach pain.
26	*Citrus aurantofolia *Christum	Rutaceae	Root powder mixed with water kills stomach worms. Fruit prevent vomiting sensation. Pounded leaves and seeds relive stomach ache in cattle.
27	*Citrus medica *Linn.	Rutaceae	Chewing dried fruit skin helps preventing dysentery. Fruit is good for indigestion. Roots are tied together along with a copper coin and placed in women's naval during child birth, which is believed to expedite the expulsion of the placenta after child birth.
28	*Citrus reticulata *Blanco.	Rutaceae	Juice by squeezing fruit skin is applied into the eyes to cure eye problems; dried fruit skin chewed to treat stomachache, tonsillitis, fever, and headache.
29	*Clematis buchananiana *DC	Ranunculaceae	Juice extracted by crushing fresh roots is inhaled, for having strong smell, to treat sinusitis and headache.
30	*Colocasia antiquorum *var. *esculenta *Linn.	Araceae	Juice of crushed roots and leaves is applied on warts. Corms are eaten as vegetable. Fresh leaves and rhizomes are used to stimulate lactation in cows.
31	*Coriandum sativum *Linn.	Apiaceae	Shoot is chewed raw to expel gas and bowel, helpful in digestion; mixed with Fenugreek and Thyme taken along with tea relieves stomach pain.
32	*Costus speciosus *Smith.	Zingeberaceae	Rhizome mixed with sugar used to treat veneral diseases; being pungent, it is used as a substitute to zinger. Juice taken before breakfast cures urinary tract infections.
33	*Cucurbita pepo *Linn.	Cucurbitaceae	Seed powder taken with water acts as vermifuge. Fresh leaf paste acts as a soothing agent if applied on the burn portion. Ripen fruits cure jaundice.
34	*Curcuma aromatica *Salisb.	Zingeberaceae	Rhizome powder taken with water relieves nausea, stomachache and expels gas.
35	*Curcuma caesia Roxb*.	Zingeberaceae	Fresh rhizome is eaten raw to expel gas.
36	*Curcuma longa *Linn.	Zingeberaceae	Drinking water boiled with root cures throat pain, cold, cough and fever.
37	*Curcuma zedoaria *Roxb.	Zingeberaceae	Fresh rhizome paste is applied externally to cure skin diseases. Rhizome eaten raw cures diarrhoea and colic, and helps in digestion
38	*Cynodon dactylon *(Linn.) Pers.	Poaceae	Crushed root juice is taken to relieve piles. Root paste applied heals cuts and wounds. Boiled leaf and root juice help in treating diarrhoea and dysentery.
39	*Daphne cannabina *Wall.	Thymelaeaceae	Root is crushed and the boiled juice is given during food poisoning. Raw leaves are fed to baby goats during diarrhoea and fever. Traditional paper is made from the bark and the stalks are used to weave mats.
40	*Datura fastuosa *Linn.	Solanaceae	In case of rabid dog bite, seed eaten raw in very minute quantity. To treat asthmatic fits, smoke from burnt leaves is inhaled.
41	*Dicentra thelictrifolia *(Wall) Hk.f & Th.	Fumariaceae	Taking water boiled with crushed root stops excessive bleeding in females.
42	*Dichroa febrifuga *Lour.	Hydrangeaceae	Leaf powder is taken during fever. Ink is prepared from berries.
43	*Dillenia indica *Linn.	Dilleniaceae	Fruit juice mixed with sugar and water is taken to treat fever. Fruit helps to relieve constipation. Leaves are used to treat dysentery.
44	*Diplazium polypodioides Bl*.	Filices	Eating fresh and dry root helps stop dysentery.
45	*Disocorea alata *Linn.	Dioscoreaceae	To relieve throat pain, rhizome is eaten raw.
46	*Drymeria cordata *Willd. ex Roem & Schult.	Caryophyllaceae	The plant is warmed while wrapped in a cloth and emanating vapour inhaled in the case of sinusitis and nose blockade. Also, it is a remedy for headache. To relive sore throat pain, fever and headache, the plant either eaten raw or cooked.
47	*Eleusine coracana *Linn.	Poaceae	Fermented seeds are taken with traditional drink as medicine during bodyache due to exhaustion. It is also given to the gastric patients.
48	*Entada pursaetha *ssp.*sinohimalensis *Grierson & Long	Mimosaceae	Juice or paste of crushed bark is applied externally to cure skin diseases. Paste of seeds is applied to cure mumps. Seed powder is mixed with water for cleansing hair, and has an anti-dandruff agent.
49	*Equisetum debile *Roxb. Ex Vaucher	Equisetaceae	Juice obtained from crushing aerial part is applied on the fresh wounds, nose bleeding etc. to clot blood.
50	*Eupatorium cannabinum *Linn.	Asteraceae	Juice obtained through crushing fresh leaves and tender shoots is applied to cuts, and the remains are placed over the wounds to stop bleeding immediately and this is highly effective in the prevention of infection further.
51	*Euphorbia pulcherrima *Linn.	Euphorbiaceae	Plant latex is applied on the toothache site to relive pain; this need great care as the latex is allergic.
52	*Evodia fraxinifolia *Hook. f.	Rutaceae	Ripe fruit is boiled to crush and the paste is applied on the forehead during giddiness; chewing raw or dried fruit treat indigestion. Fruits are also used to make chutney.
53	*Fagophyrum esculentum *Moench	Polygonaceae	Powdered grains are baked into chapattis (Bread) and given to treat diarrhoea.
54	*Ficus cunia *Ham.	Moraceae	The latex is applied externally to reduce boils.
55	*Ficus hirta *Vahl.	Moraceae	Root decoction treats food poisoning.
56	*Ficus religiosa *Linn.	Moraceae	Water extract of any plant part is given during burning sensation of the genitals. Bark soaked in water and the water is taken to stop vomiting. Fruit juice is used in to treat cracked feet.
57	*Gouania leptostachya *DC	Rhamnaceae	Past of leaves is applied to cure sores and inflammation.
58	*Helianthus annus *Linn.	Asteraceae	Root decoction as a gargle relieves toothache; dried flower chewed cures ulcers, fever, cough and cold. Leaves crushed and mixed with water and taken bath cures Allergy and skin diseases are treated taking bath with leaves crushed into water.
59	*Heracleum wallichii *DC.	Apiaceae	Dried fruits are chewed to treat sinusitis and influenza. Root juice is taken to cure diarrhoea; seeds are locally used as chatni.
60	*Hibiscus esculentus *Linn.	Malvaceae	Fruit mucilage acts as soothing agent on cuts.
61	*Holarrhena antidysenterica *Wallich	Apocynaceae	Powder of barks, seeds and leaves is taken with water helpful in in dysentery.
62	*Hordeum vulgare *Linn.	Poaceae	Gruel is made by the powdered grains and given in case of painful indigestion. Barley water with honey is prescribed in bronchial coughs.
63	*Hydrocotyle asiatica *Linn.	Apiaceae	Fresh plant parts crushed and ingested orally cure sores of throat and lungs. Leaf juice is used as eye drops to cure eye infection. Dressing with leaf paste reduces swelling or and applied in wounds. Juice of shoots treats gastritis and constipation.
64	*Juglan regia *Linn.	Juglandaceae	Fresh bark juice is taken to remove worms from the stomach. Bark and leaves crush act as a fish poison. The nuts are eaten. The shell of the fruit when crushed gives out black color which was used previously to paint the door and the windows.
65	*Kaempferia sikkimensis *(King ex Baker) K. Larsen	Zingeberaceae	Poultice formed from crushed bulbs is applied to heal bone fractures, dislocation and wounds.
66	*Lantana camara *Linn.	Verbinaceae	The juice of crushed leaves is applied to the fresh cut and wounds to heal. Crushed leaves are tied over the sprain to relieve pain.
67	*Leea macrophylla *Roxb.	Leeaceae	Seeds are wrapped, as small pack, in a cloth and tied around the neck of the children, which is believed to cure stomach pain. Also, the seeds are chewed to treat viral fever.
68	*Lindera neesiana *(Wall ex Nees) Kurtz.	Myrsinaceae	Seeds crushed and taken with water stops vomiting sensation.
69	*Litsea citrata *Blume	Lauraceae	Fruits are chewed to treat stomach disorders, headache; also used in making chutney.
70	*Lobelia angulata *Forst.	Lobeliaceae	Whole plant is boiled and given in case of throat pain and fever. Tender shoot is smashed and the juice is applied externally to treat boils and inflammation.
71	*Luffa aegyptiaca *Mill. ex Hook. f.	Cucurbitaceae	Juice of leaves cures conjunctivitis. Tender fruit is taken as vegetable. The course sponge of mature fruit is used as a bath scrub.
72	*Lycopersicon esculentum *Mill.	Solanaceae	Raw fruit is taken during indigestion and to prevent bleeding from the gums.
73	*Marsdenia roylei *Wight.	Asclepiadaceae	Decoction of unripe fruit, root and leaf is to relieve burning sensation in the genitals.
74	*Mentha arvensis *Linn.	Lamiaceae	Raw leaves chewed help to check stomach related disorders: gastritis, acidity, indigestion etc., also used to flavour chutney.
75	*Mimosa pudica *Linn.	Mimosaceae	Decoction of roots is helpful to control piles; root paste is applied externally to cure boils.
76	*Momordica charantia *Linn.	Cucurbitaceae	Fruit juice is good for diabetics; juice acts as blood purifier. Juice of tender shoot or root is applied at the point of snake bite.
77	*Morus indica *Linn.	Moraceae	Bark and leaf decoction cures sore throat; fruit is edible and cures throat infection and swelling. Seed extract is applied to heal foot cracks.
78	*Mucuna marcrocarpa *Wallich	Fabaceae	Seed powder taken with water helps remove round worm from stomach.
79	*Musa paradisiacal *Linn.	Musaceae	Person suffering from fever is advised to drink sap released from the plant directly.
80	*Mussaenda frondosa *Linn.	Rubiaceae	Whole plant is boiled and decoction is given to treat fever, asthma and cough.
81	*Nasturitium officinale *R. Br.	Brassicaceae	The aerial part decoction is given to relieve body pain; also eaten as salad.
82	*Oroxylum indicum *(L.) Kurz	Bignoniaceae	Bark and seeds are powdered and mixed with water, and strained; the concoction is fed to patients suffering from high fever or pneumonia, which believed to restore health or brings down fever. Unbroken pod is also used in rituals.
83	*Oxalis corniculata *Linn.	Geraniaceae	Whole plant is chewed raw and the juice acts as an appetizer; also checks boil. Fresh plant decoction taken treats dysentery. Fruit is consumed to lessen throat pain.
84	*Paederia scandens *Merrill	Rubiaceae	Dried fruit is powdered and applied over teeth to relieve tooth ache and prevent tooth decay.
85	*Pandanus nepalensis *St. John	Pandanaceae	Tying or wrapping up the young and tender leaves from upper part of the stem on the surface act as an antidote to snake poison/bite. It may also be chewed as breath sweetener. Fresh leaves act as cockroach repellant. Leaves are used for making mats, carry bags, fishing bags and for thatching purpose. Fruits are seen being eaten by monkeys and rats.
86	*Phyllanthus emblica *Linn.	Euphorbiacea	Fruit is eaten raw to treat cough, diarrhoea, and dysentery.
87	*Phytolacca acinosa *Roxb.	Phytolaecaceae	Fresh leaves are boiled and consumed to relieve bodyache and diarrhoea.
88	*Picrorhiza kurroaa *Benth.	Scrophulariceae	Dried rhizome is boiled in water and taken to cure fever, cough, etc.
89	*Pieris ovalifolia*	Ericaceae	Leaves either crushed or mixed with water are rubbed on the body to reduce inflammation, irritation and allergies.
90	*Piper longum *Linn.	Piperaceae	Dried seed powder paste is applied to reduce sprains; the powdered roots are given to treat cold and cough.
91	*Plantago eroasa *Wallich	Plantaginaceae	Leaf paste is applied to heal wounds. Seed powder is taken with water treats diarrhoea and dysentery.
92	*Polygonum viviparum *Linn.	Polygonaceae	Root juice boiled with water is given in case of fever and stomach upset.
93	*Prunus cerasoides *D. Don	Rosaceae	Bark is powdered and applied externally on the fractured bone along with other processs of treatment; fruit is edible.
94	*Psidium guajava *Linn.	Myrtaceae	Young leaves and tender shoots taken raw cure mouth ulcers, sore throat, cough, toothache. Drinking bark powder mixed in hot water is best local remedy for dysentery with blood in stool; fruits are edible.
95	*Pteris biaurita*	Pteridaceae	Mashed petiole extract applied on the cuts and wounds stop bleeding and infections.
96	*Rhododendron arboreum *Smith	Ericaceae	Dried flowers crushed and mixed with water stop excessive bleeding in female. Fresh leaves chewed stop dysentery. Flower petals clear throat choking due to fish or chicken bone.
97	*Rhododendron campanulatum *D. Don	Ericaceae	Leaves are chewed and the juice from the crushed leaves relieves cough.
98	*Rhus semialata *Murr.	Anacardiaceae	Sour juice of fruits is boiled with water, and concentration is further mixed with water and raw egg, treats diarrhoea and dysentery. It is also used as food preservative.
99	*Rubia cordifolia *Roxb. ex Fleming	Rubiaceae	Root decoction with water is given to cure urinary infection; paste is used as an ointment to skin diseases. Root is also used to make dyes.
100	*Rubus ellipticus *Smith	Rosaceae	Young shoot is chewed raw to relieve sudden stomach pain. Root decoction given to the children to get rid of stomach warm. Root paste is applied on forehead during severe headache; fruit is edible.
101	*Rumax nepalensis *Sreng.	Polygonaceae	Juice prepared by smashing leaves and young shoots is applied to heal wounds. Root is crushed and the juice applied on the scalp prevents hair loss.
102	*Saccharum officinarum*	Poaceae	Juice is taken to cure jaundice.
103	*Sapindus mukorossi *Gaertn.	Sapindaceae	Scalp is washed with fruit to remove dandruff and lice.
104	*Schima wallichii *(DC.) Korth.	Theaceae	Bark is rubbed on the caterpillar infected portion removes its hair.
105	*Semecarpus anacardium *Linn. f.	Anacardiaceae	Root paste (poisonous) is applied externally on the affected portion cures skin diseases. Decoction of the bark is given to the animals to treat worms.
106	*Solanum khasiana *C.B. Clarke.	Solanaceae	Smoke, through burning the seeds, is directed to the infected teeth to cure toothache and tooth decay.
107	*Spermadictyon suaveolens *Roxb.	Rubiaceae	Root paste is applied externally to relieve joint pain.
108	*Sphagnum squarrosum *Crome	Sphagnaceae	Hunters and graziers use whole moss for dressing wounds in place of absorbent cotton or gauze. It is also act as an important source of fuel for them.
109	*Stephania hernandifolia *Walp.	Minispermaceae	Paste of crushed leaves is applied on the boils for opening; water kept in bulbous root is sprinkled in the poultry farm to prevent from bird flu.
110	*Swertia chirayita *(Roxb. Ex Flem.) H. Karst.	Gentianaceae	Juice obtained through boiling the entire plant is taken to cure fever, cold, cough, diarrhoea, and stomach-ache.
111	*Thysanolaena maxima *Kurtz.	Poaceae	Root paste applied on boils helps it in opening up faster. Juice from boiled roots used as gargle in case of bad breath and kills worms in stomach on drinking. Broom and roots are tied together along with a copper coin and placed in women's naval during child birth, believed to expedite expulsion of the placenta after child birth. During wedding rituals and Pujas (Prayers) for newly constructed houses, individual stalks or bouquet are placed in several locations around the house to create an auspicious environment.
112	*Tupistra nutans *Wall.	Liliaceae	Inflorescence is powdered and mixed with water and taken to relieve body pain.
113	*Usnea sikkimensis*	Parmeliaceae	Hunters and graziers use it to bandage surface wounds and skin eruptions or boils. It is inserted in the nostril to stop nose bleeding. Shepard put it in the shoe to prevent or treat blisters.
114	*Urtica dioica *Linn.	Urticaceae	Root paste is applied on minor bone fracture and dislocation. Root and seed decoction is taken to treat diarrhoea and cough. Curry, prepared using shoot tips, is given to female during child delivery as their slipperiness is believed to help delivering child. It should not be taken by a person who has been bitten by rabid dogs which is believed to aggravate the problem. Stems are beaten, dried and boiled to make threads and woven into traditional nettle clothing. Spines believed to stimulate milk production, when cows do not lactate, they are believed to be possessed and beaten with nettles for normal lactating. Shamans beat humans during exorcism rituals with nettles in a belief to drive away evil spirits from body; this should not be touched or eaten by family members of deceased person on the day of death. If the decease is one's father or mother, this prohibition remains for one year. Nettle is planted on the child's grave in a belief that the evil spirit of child will not come out to trouble other family members.
115	*Valeriana hardwickii *Wallich	Valarianaceae	Extract of crushed roots is taken to treat urine trouble.
116	*Viscum articulatum *Burm.f.	Loranthaceae	Paste prepared from the entire dried plant is applied to heal fractured bone, and dislocation.
117	*Zanthoxyllum alatum *Roxb.	Rutaceae	Branchlet used as toothbrush to relieve toothache. Berries (2–3) taken to cure stomach ache and toothache. Berries are crushed and rubbed on the leg which acts as leech guard.
118	*Zingiber officinale *Rose.	Zingeberaceae	Rhizome is roasted and chewed to treat cough, fever and throat problem.

**Figure 3:**
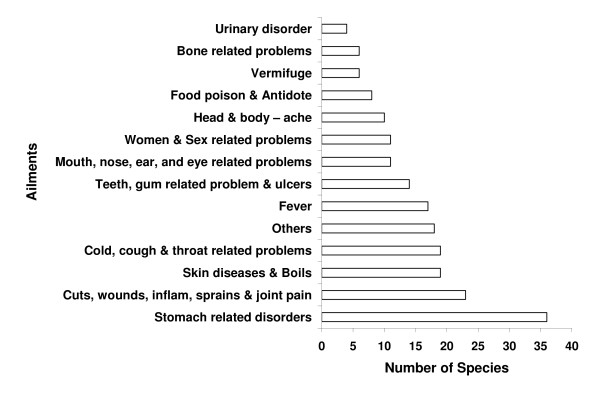
Major group of ailments cured using the plant species in Dzongu valley, North Sikkim, India.

The 118 medicinal plant species recorded from Dzongu are used to cure about 66 ailments, which authors grouped them under 14 broad categories (Figure 3). Of which, 36 species (maximum) used to treat stomach related disorders such as diarrhoea, dysentery, indigestion, gas expelling and others; however, 23 species figured in curing cut, wounds, inflammation, sprains and joint pain (Figure 1). The study revealed that 59.3% plant species are reported to be used to cure more than one ailment. External applications as well as internal consumption are involved in the treatment of diseases. Analysis of species level data discovered the oral (75.0 %), external application (44.4%), nasal (5.5%), eye (2.7%) and the ear (0.93%) as major administration route of ethnomedicine used. It was observed that most of the preparations include single plant species and in rare case the combination of two or more species. It was also observed that different parts of a single species are used to cure different diseases.

The study finds the used administrations are not standardized in general, but depend on the age and physical appearance of the patient, illness and diagnosis of the diseases [28,42,43]. Children are given small doses of medicine than considered in case of adult patients, which further depend on the type of illness and treatment realized appropriate by the local medicine man. The type of disease and level of its severity further decide the course of the frequency of treatments. Each medicinal plant is used either raw or in dried form as medicine. Especially, the underground parts are used in the dried form, which is either cut into small pieces or powdered, and stored [44].

On the collection and use of medicinal plants, about 70% respondents indicated *Swertia chirayita *as the most frequently used and highly extracted species (whole plant) for its applicability in many common diseases, such as, fever, cold, cough, diarrhoea, stomach-ache (Table 2). As per IUCN criteria the *S. chirayita *is considered as a critically endangered species for Himachal Pradesh, India [45] and vulnerable for north-east India [46]. Similarly, the crushed rhizome of *Bergenia ciliata*, a threatened medicinal herb [46] is used to cure fractured bone, fresh cuts and wounds. Whereas, in west Sikkim, graziers also use the same in case of sheep, Dzos (a breed of Ox and Yak) and horses (authors' unpublished work). An endangered species for both Sikkim and Arunachal Pradesh [46], the *Aconitum ferox *is a poisonous plant and has traditional use for fever, skin diseases, cough and gout. There are many threatened medicinal plants grow along the high altitude reaches of Dzongu, such as *Aconitum heterophyllum, Dactylorhiza hatagirea, Nardostachys jatamansi, Panax pseudosinseng, Picrorhiza kurrooa*, etc. These species were used for ethnomedicine in the past but owing to distance of availability, severely declined populations and loss of knowledge amongst youngsters, the majority of respondents did not mention them as under current use. Interestingly, the *A. heterophyllum, N. jatamansi *are assessed as endangered and *P. kurrooa *as vulnerable under IUCN criteria for Sikkim [46].*Oroxylum indicum *is yet other vulnerable (IUCN criteria) taxa for Sikkim, having common utility for folk as appetizer and to treat dysentery and throat pain. Another destructive use by extracting roots in urine trouble in case of vulnerable species for Sikkim, *Valeriana hardwickii *is known. *Dioscorea alata*, a common form of wild edible for Sikkim people is also found to be used, occasionally, for having medicinal properties, in curing fever, rash, itches, constipation and piles.

Use of *Pandanus nepalensis *as medicine is poorly mentioned in the literature though has important properties. This plant is abundantly available all along the Teesta valley and its tributaries in the warmer parts of the state, including lower parts of Dzongu. Belonging to monocot family it is a medium-sized tree up to 5–6 m in height typically having broad canopy and stout trunk, ringed with many leaf scars and dioeciously branched. The clustered drooping fruit resembles *Ananas comosus*, but without leaves at the apex of the fruit, which is seen eaten by monkeys and rats. As per Moan-doak, placing or tying up of young or tender leaves on the skin at the place of snake bite helps reduce the pain caused. It may also be chewed as breath sweetener. The fresh leaves also act as a cockroach repellant. The leaves were used for making hand-wooven mats, carrying bags, fishing bags, thatching roofs, etc. but a dying practice these days.

The use of *Sphagnum squarrosum *(peat moss) and *Usnea sikkimensis *(old man's beard, a lichen) of the alpine region, in dressing and bandaging cuts and wounds because of their absorbency and insulation, has been reported by some of the elderly persons, who were the hunters and graziers at one time. *S. squarrosum *is also used as an important source of fuel in the area. Written records exist on the use of *U. sikkimensis *as a remedy for lung troubles, hemorrhage and asthma, and also the massaging scalp with plant powder helps strengthen hair [14]. Thyongs of Dzongu also reported that *U. sikkimensis *stops nose bleeding, prevents or treats foot blisters (if inserted inside the shoe) due to continuous wearing of hard leather shoes, and treats skin eruptions and boils (bandaged over the wound). This lichen is inserted in a bag and also used in the form of pillow by the graziers/shepherds. However, such uses of plants sound amazing and interesting to the present generations.

## Discussion

In general, over 80% respondent under present study in Dzongu shared that in recent years, dependency on allopathic treatments has increased considerably over traditional health care systems. Loosening interest amongst in young generation, and tough and time consuming process of plant collection and gradually lacking in skill of specific identification, appeared as major reasons for declining trend in using traditional health care system. For living in the close nearness to the district headquarter by the exposures and involvement in developmental programmes offering them livelihood options as well the availability of primary health centers and sub-centers in each village in recent years have further diverted youngsters from using ethnomedicinal practices. Surprisingly, for some particular ailments like bone fracture and dislocation, most of the inhabitants still prefer herbal use rather than the allopathic treatment, as they like to avoid undergoing painful therapies of the later. Many natives still prefer and trust upon using traditional health care system as the excellent and much effective means to cure their ailments over allopathic drugs [42-44]. The species subjected to destructive harvesting due to uprooting underground part form over 29% in Dzongu. Often, the threatened taxa, if they are already having small and fragmented populations in a particular area, as well as growing in specific habitats [41], they could be susceptible to further endangerment, if species are approached to commercialization through wild harvesting. It would be crucial to assess their potential of availability, as resource, through population assessment. Ex-situ cultivation of such taxa would not only promote their conservation but also offer income opportunity to local folk. Amongst them, some, including high traded threatened taxa *Swertia chirayita *and *Picrorhiza kurrooa*, are prioritized at the top for their conservation through ex-situ cultivation [12].

Prior to entering Sikkim from southwest Tibet, the Lepcha tribe migrated to Thailand, Burma, Assam, and Bhutan. During the course of migration, they got along the composite culture over how to use the available wild plants of those areas and importantly the knowledge of those herbal plants associated with well being of mankind and deeply in them efficiency of the drug's crucial for saving life. In turn in Sikkim, they encountered many new plant species and developed their knowledge on them. They decided "*Ne Mayal Lyang*", on the slopes of Khangchendzonga (floristically rich) in Sikkim as their final abode. From their experience in the past new discovery left them rather to experiment the new plant species for different ailments in addition of plants as medicine in the number. It seems that Lepcha tribe of Dzongu valley was a keen learner over the use of plants for their property of drug through experience and natural selection not been possessed by other and hence decided to keep their knowledge upto themselves in the threat of life as a survival strategy. This has made them most experienced medicinal practitioner and to the community a container of those associated culture. During authors' latest conversation with one of the elderly Lepcha from Dzongu, he mentioned that the cut piece of dried rhizome of *Acorus calamus *is given to child for speech clarity or to the stammering a child, and has been found effective in curing the problem, which is a new finding for Lepcha tribe. The Apatanis uses the root/rhizome of the same species for curing problems like cuts, wounds, skin diseases, bone fracture but Lepcha uses it for curing cough and fever in addition to skin diseases (Table 1). But they do not use it for cut, wounds, bone fracture etc. because they found *Bergenia ciliata *to be much more effective in case of such problems and *Viscum articulatum *in case of bone fracture than *Acorus calamus*. Similarly, *Ageratum cornyzoides *is used by the Lepcha tribe for curing diarrhoea, dysentery, intestinal colic with flatulence in addition to cut and wounds as used by the Apatinis and the Jaintia tribe of the North-eastern India. Similarly, the use of *Allium cepa *is different for Lepcha tribe and the Apatani tribe (Table 1.). Lepchas have learnt to make use of *Costus speciosus *for curing the disease infecting most sensitive part of the human body (veneral disease and the urinary tract infection), which is not mentioned by other tribes [47-50] under review (Table 1). Similarly, the leaf of *Lantana camera*, the dominant weed in the region, is being found used only by the Lepchas as an antiseptic and as a pain reliever; this use is not found with other tribes mentioned in this paper. Depending upon the immediate availability of the plant species, they have managed to make multiple uses of single species. For example, *Urtica dioica*, is used by the Lepcha tribe for curing diarrhoea and cough and the soup prepared from it is given to the pregnant women which helps is easy delivery of child other than bone fracture as used by Apatani tribe. Similar multiple use of another species, *Cynodon dactylon, Drymeria cordata *and *Ficus religiosa*, is recorded form Lepcha tribe of Dzongu (Table 1), such use is not reported from other tribes of the north-eastern Indian region indicating that the Lepchas having much more exploratory power and knowledge in comparison to the other existing tribes in the region.

Use of local medicinal plants by Lepchas ensures the continuity of indigenous knowledge associated with the species and has the definite bearing on the identification of their habitats, which are confined in the pockets of the most difficult hill terrain to some extent. The gradual decline in traditional use practices may, therefore, leads to the fading away of the indigenous knowledge associated with the plants in very near future. On the other hand, the people inhabiting Sakyong- Pentong, Tholung, etc., the places which are not approachable by roads, still found to be almost fully dependent on herbal health care system. The present study indicates that the Dzongu area is a rich reservoir of medicinal plants and associated ethnomedicinal practices offering great pharmaceutical potential. The knowledge for identification of medicinal plants, drug preparation and usage for medicines, as great potential amongst Lepcha tribes of Dzongu valley is confined to few old traditional practitioners chiefly. For their getting migrated to cities in search of better livelihood options further weaken the interest of young generations in carrying noble traditions. This tendency of disinterestedness in old traditions is feared by old generation as a major cause of loosing this wealth of knowledge in coming time soon. Therefore, it is an appropriate time to document systematically traditional ethnomedicinal practices for conservation.

Introducing techniques of ex-situ cultivation of commercially viable species [12,51,52] would present a strong option of income generation to community people. To establish self sufficient primary health care system of this remotely placed tribal area, growing herbs in kitchen garden would not only supply raw material at household level but ensure the revival of traditional knowledge and conservation of valuable medicinal plants of the region. Development of kitchen garden growing herbs has greater benefit to train community tribal people on conservation through nursery practices at small scale before venturing into big ones. The current study may be of great use and interest to researchers, pharmaceuticals, foresters and medicinal practitioners. The documentation finds Dzongu valley a highly potential reservoir of high value medicinal plants and rich ethnomedicinal knowledge, and can also be a suitable agroclimatic zone for the cultivation of herbal plant species. Thus the current study will further help in both conservation of traditional ethnomedicinal knowledge as well as the development of native villagers.

## Competing interests

The authors declare that they have no competing interests.

## Authors' contributions

The current study is a joint effort of both authors. BKP collected data, relatively, for a longer period in field, computed them for statistical analysis and contributed in primary manuscript drafting. HKB conceptualized and designed the study; collected field data, interpreted them and finalized the draft. Both authors read and approved the final manuscript.
